# Development of a Core Outcome Set and Minimum Reporting Set for intervention studies in growth restriction in the NEwbOrN (COSNEON): study protocol for a Delphi study

**DOI:** 10.1186/s13063-019-3588-9

**Published:** 2019-08-17

**Authors:** Martine Knol, Helena Wang, Frank Bloomfield, Tabitha Piet, Stefanie Damhuis, Asma Khalil, Wessel Ganzevoort, Sanne Gordijn

**Affiliations:** 1 Department of Obstetrics and Gynecology, University Medical Center Groningen, University of Groningen, Groningen, The Netherlands; 2 Liggins Institute, University of Auckland, Auckland, New Zealand; 3 Fetal Medicine Unit, St George’s University Hospitals NHS Foundation Trust, University of London, London, UK; 4 Vascular Biology Research Centre, Molecular and Clinical Sciences Research Institute, St George’s University of London, London, UK; 5 Department of Obstetrics and Gynecology, Academic Medical Center, University of Amsterdam, Amsterdam, The Netherlands

**Keywords:** Growth restriction in the newborn, GRN, Fetal growth restriction, FGR, Intervention, Core outcome set, COS, Minimum reporting set, MRS

## Abstract

**Background:**

Growth restriction in the newborn (GRN) can predispose to severe complications including hypoglycemia, sepsis, and necrotizing enterocolitis. Different interventions and treatments, such as feeding strategies, for GRN have specific benefits and risks. Comparing results from studies investigating intervention studies in GRN is challenging due to the use of different baseline and study characteristics and differences in reported study outcomes. In order to be able to compare study results and to allow pooling of data, uniform reporting of study characteristics (minimum reporting set [MRS]) and outcomes (core outcome set [COS]) are needed. We aim to develop both an MRS and a COS for interventional and treatment studies in GRN.

**Methods/design:**

The MRS and COS will be developed according to Delphi methodology. First, a scoping literature search will be performed to identify study characteristics and outcomes in research focused on interventions/treatments in the GRN. An international group of stakeholders, including experts (clinicians working with GRN, and researchers who focus on GRN) and lay experts ([future] parents of babies with GRN), will be questioned to rate the importance of the study characteristics and outcomes in three rounds. After three rounds there will be two consensus meetings: a face-to-face meeting and an electronic meeting. During the consensus meetings multiple representatives of stakeholder groups will reach agreement upon which study characteristics and outcomes will be included into the COS and MRS. The second electronic consensus meeting will be used to test if an electronic meeting is as effective as a face-to-face meeting.

**Discussion:**

In our opinion a COS alone is not sufficient to compare and aggregate trial data. Hence, to ensure optimum comparison we also will develop an MRS. Interventions in GRN infants are often complicated by coexisting preterm birth. A COS already has been developed for preterm birth. The majority of GRN infants are born at term, however, and we therefore chose to develop a separate COS for interventions in GRN, which can be combined (with expected overlap) in intervention studies enrolling preterm GRN babies.

**Trial registration:**

Not applicable. This study is registered in the Core Outcome Measures for Effectiveness (COMET) database. Registered on 30 June 2017.

**Electronic supplementary material:**

The online version of this article (10.1186/s13063-019-3588-9) contains supplementary material, which is available to authorized users.

## Background

Growth restriction in the newborn (GRN) is the post-partum equivalent of fetal growth restriction (FGR) [1]. Optimizing care of the GRN may positively influence the degree of adverse outcomes in the long-term. Many interventions and treatments for neonates born with growth restriction are symptom-driven, such as for hypothermia and preterm birth. Currently, the most common specific interventions for GRN are feeding strategies. Optimized postnatal feeding strategies are aimed at enhancing growth and reducing the incidence of short-term complications such as hypoglycemia, sepsis, and necrotizing enterocolitis (NEC) [2, 3] and long-term neurodevelopmental outcome (including IQ). Positive immediate effects (faster growth) may, however, have a potential negative trade-off for metabolic health (obesity, cardiovascular risk) [4].

Different interventions and treatments have their own/specific benefits and risks [2, 3, 5]. Comparing the results from these studies is challenging due to the use of different baseline and study characteristics and also because of differences in reported study outcomes [6]. In order to be able to compare study results and to allow pooling of data, uniform reporting of study characteristics (minimum reporting set [MRS]) and of outcomes (core outcome set [COS]) are needed.

An MRS considers the study and population (baseline) characteristics that should, as a minimum, be reported, such as the study population, the details of the intervention, the references and what exactly is under study. An MRS for FGR has been developed [7], but this has not yet been performed for studies in GRN. A COS is a minimal set of outcomes that needs to be measured on a certain topic [8]. A COS for FGR also has been developed [9], but this has also not yet been performed for interventions and treatments for GRN.

We aim to develop both a COS and an MRS for GRN using the Delphi methodology according to the COMET initiative [8–10]. Their development will be informed by the existing COS and MRS for FGR [7, 9]. Medical experts (obstetricians/gynecologists and neonatologists), researchers, and parents will be involved in the consensus process, in line with the recommended methodology of the COMET initiative. Parent (lay expert) involvement in the whole process of development and procedure of the COS and MRS ensures that they include relevant outcomes and items for the target population. A potential barrier for implementation could be too many outcomes included in the final COS, which would make implementation more difficult. We try to avoid this by stressing exclusivity rather than inclusivity during the Delphi rounds.

### Objective

The objective is to develop an MRS and a COS for interventional and treatment studies in GRN.

## Methods/design

### Overview

**Fig. 1:**
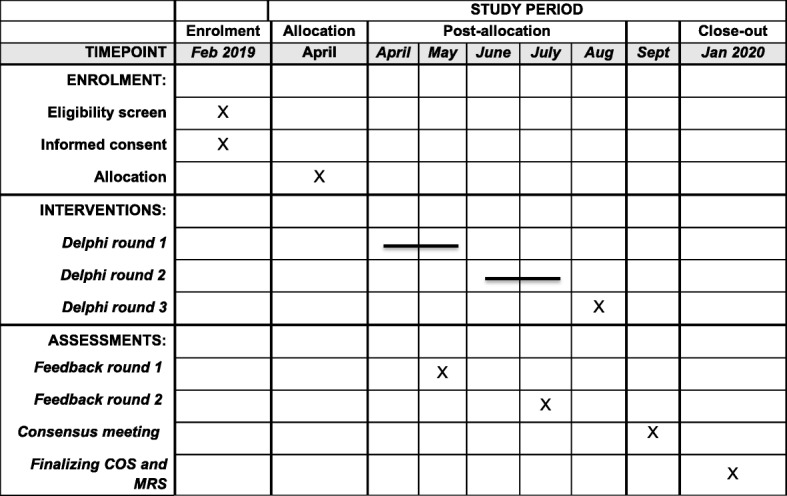
SPIRIT figure. This figure depicts the timeline of COSNEON

To develop the Core Outcome Set and Minimum Reporting Set for intervention and treatment studies in growth restriction in the NEwbOrN (COSNEON) a stepwise approach will be used according to the COMET handbook, with some slight amendments [8]. This study will start in February 2019, with an expected date of completion in January 2020 (Fig. 1). Our study is registered at the COMET initiative, accessible via http://www.comet-initiative.org/studies/details/1001?result=true. This protocol was checked via the SPIRIT checklist (Additional file 1) and via the COS-STAP checklist (Additional file 2).

### Scope of COS determined

The target population for the COS will be growth restricted babies, either diagnosed antenatally (FGR) or postnatally (GRN). The international consensus definitions of FGR and GRN both apply [1, 10]. We want to develop a single COS for GRN, focused only on interventions for growth restriction and not considering preterm birth, a frequent comorbidity, as a COS has already been developed [11], nor considering prevention as this is captured by the COS for FGR.

### Determine what to measure

First, we will conduct a scoping search strategy to identify study characteristic items and outcomes reported in intervention and treatment studies following growth restriction in the newborn in the last five years. The search will be performed in the database PubMed (search strategy: ((Diet [tiab] OR Nutritional management [tiab] OR Feeding [tiab] OR Diet therapy [tiab] OR “Nutrition Therapy”[Mesh] OR “Therapeutics”[Mesh] OR “Treatment Outcome”[Mesh] OR therapeutic* [tiab] OR treatment* [tiab] OR treatment outcome* [tiab] OR intervention* [tiab]) AND (small for gestational age [tiab] OR intra uterine growth restriction [tiab] OR IUGR [tiab] OR Very low birthweight [tiab] OR SGA [tiab] OR Fetal growth restriction [tiab] OR FGR [tiab] OR Growth restricted fetuses [tiab] OR Fetal growth retardation [tiab] OR “Fetal Growth Retardation”[Mesh] OR “Infant, Low Birth Weight”[Mesh] OR growth restricted newborn [tiab] OR GRN[tiab])) Filters: Clinical Trial; published in the last 5 years; Humans). For each relevant article we will list the study details, baseline characteristics, and primary/secondary outcome(s), author, title, and publication year. We will only assess interventional trials and no observational studies.

#### Inclusion criteria

- Studies concerning GRN and interventions or treatments
- Interventional trials
- The study is published in the last 5 years

### Delphi survey

For development of the MRS and COS we will use the Delphi methodology, an iterative process that narrows down opinions until consensus is reached on a certain scientific domain. Several stakeholder groups will be approached in three survey rounds using RedCAP version 7.3.2. The first stakeholder group comprises the lay experts consisting of parents of infants with GRN and parents who are expecting GRN (the fetus is diagnosed as FGR). The second and third groups of stakeholders consist of professionals. We want to include pediatricians, neonatologists, obstetricians/gynecologists, and other health professionals whose work focuses on GRN in the second group and researchers with focus on babies with GRN in the third group. In the Delphi method a list of study characteristic items and outcomes is proposed to all participating stakeholders (the ‘participants’). These are rated for relevance using the Likert scale (1, really unimportant; 5, really important). After each round the aggregated results are presented to the participants, who are then asked to reconsider their score in light of the majority opinion. On average, two or three rounds are needed to reach consensus [12]. After the third round, there will be a final consensus meeting to reach consensus on the MRS and COS.

### Recruitment of participants

For the development of the MRS and COS we will include the previously described experts in the area of intervention and treatment studies in GRN babies. Selection of the experts will be by authorship on the topic of fetal growth restriction/growth restriction of the newborn and interventions, by use of a known group of experts who have been involved in other Delphi procedures, and by asking participants to mention other possible experts in the field. The email will contain a digital flyer with a very brief explanation and an email address and QR link and URL that links to a more detailed invitation to participate in the study including an explanation of the study purpose and details regarding privacy handling (Additional file 3). Moreover, we will produce printable flyers with the QR code, email, and URL to be disseminated at meetings and conferences. The approach will be in line with privacy regulations and participants have the option to withdraw at any time.

For recruiting parents of GRN babies we will use an indirect approach. We will have posters and flyers inviting parents to participate at the obstetric/gynecology, pediatric outpatient clinics, NICUs, at midwifery clinics, and at different GP practices located in Groningen, Amsterdam, London, and Auckland. Furthermore, patient advisory groups will be asked to share the study details and the posters through social media and with their international patient advocacy contacts. The investigators’ institutional social media accounts will also be used to advertise the study. Participants must have adequate English skills and lay experts will be involved in the wording of the posters and email. We aim for at least 50 parents completing the procedure. Before and after each round we will stress the importance of completing the entire Delphi survey to ensure good reliability of the results.

### Proposed study items and outcomes

The outcomes and study items reported in the studies identified in the search will be presented to the expert panel. Additional outcomes/study characteristics that have been selected for the COS and MRS of GRN and apply to the postnatal period are also presented to the panel with the suggestion to include these in this COS as well.

### Involvement of patient and public

Parents will participate in the Delphi survey procedure, both in the development of the protocol, design of the procedure, and in the consensus meeting.

### Data collection and analysis

We anticipate a three round Delphi procedure. Each subscribed participant will receive an email with a link to the online questionnaire. In subsequent rounds the results of the former round will be presented to the participants. The results will be presented aggregated at a group level and no individual answers will be reported. In subsequent rounds the participants are asked to rate the importance of the presented aggregated outcomes taking the results of the former round into consideration. If a participant fails to respond in a round, he/she will not be invited to the subsequent round. We will send reminders if a participant does not respond in two weeks and a final reminder two days before the deadline. Each round will be open for three weeks.

#### First round

The subscribed participants will be invited to participate by an email with a link to the first round in RedCAP version 7.3.2. They will be asked to score the candidate study characteristic items and outcomes for their relevance. A five-point Likert scale will be used, slightly different from the COMET Handbook. A five-point scale rather than a seven- or a nine-point scale is chosen for a number of reasons proven from literature, one being that the response rate is higher using a five-point scale than a seven- or nine-point scale because it is easier and quicker [13]. The questions will be grouped into MRS and COS domains. A median score of 5 is the predefined criterion for the outcome to be part of the COS/MRS. Additionally the participants are asked if, in their opinion, any important outcomes were missed, to ensure that all important outcomes are included in the MRS and COS. All additional outcomes suggested by at least two participants will be discussed in the steering group for inclusion in the subsequent round. We will ask the participants whether they will possibly be available for a consensus meeting, with confirmation not essential at this point in time. In case they are willing to participate we will provisionally ask them if they will be available to attend a meeting taking place in Maastricht 17–21 of September 2019 either in person or online through electronic media.

#### Second round

The results of the first round will be presented in the second round. The median of each outcome will be presented per stakeholder level, visible for all participants regardless of the stakeholder group to which the participant belongs. The participant will be asked to re-score the outcomes, now with the knowledge of the viewpoints of the other expert stakeholder groups. Again, we will ask for possible participation in a consensus meeting.

#### Third round

This is the final round before the consensus meeting. Results of the previous round will again be presented at stakeholder level. In this round we ask whether the experts agree to accept the outcomes with a median Likert of 5 after round two and whether they agree to the rejection of outcomes with a median Likert less than 4. The predefined level of agreement is 70%. Participation in a consensus meeting will now have to be confirmed; all participants at the consensus meeting will be acknowledged in the manuscript (after agreement).

### Consensus

To finalize COSNEON we will organize two separate consensus meetings to answer a pressing methodological matter regarding Delphi procedures in general. At this point the gold standard is a face-to-face meeting. Considering the limited funding options for COS and the ideal of including international experts and lay experts, we are interested in the option of an electronic meeting. We aim to investigate whether an electronic consensus meeting results in the same included variables in the COS and MRS as a face-to-face meeting. We aim to do this by organizing one electronic meeting with at least three representatives of all groups, and a face-to-face meeting as a satellite meeting of the third Congress of joint European Neonatal Societies (jENS) on the 17–21 September in Maastricht. All outcomes still included in the list after round 3 will be taken forward for voting. Each stakeholder group is of equal importance.

## Discussion

We plan to develop both an MRS and a COS for intervention studies in GRN. In our opinion a COS is not sufficient to compare and aggregate trial data. By only defining the end output of the trials without properly defining the inputs, the quality of the end product cannot be assessed fully, only the appearance of it. We acknowledge the fact that interventions in GRN infants are often complicated by delays in achieving full enteral feeds because of gastrointestinal immaturity and consequent feeding intolerance. When enteral feeding is not possible, parenteral feeding is an alternative. However, this carries risks of central-line associated bloodstream infections and also intestinal failure-associated liver disease in the case of prolonged parenteral feeding [14]. These outcomes are highly associated with preterm birth for which a COS has been developed previously [11]. Therefore, we chose to develop a COS for interventions in GRN separately, which is to be combined (with expected overlap) in studies for preterm GRN babies.

We will disseminate the COS and MRS via appropriate media and during relevant international meetings. The relevance of the COS will also be discussed with patient/parent organizations. It is vital that the COS and MRS will be used in future research which focuses on the GRN. This will increase the relevance of these studies and it will simplify comparing them.

## Additional files

Additional file 1: SPIRIT checklist. (PDF 99 kb)

Additional file 2: COS-STAP checklist. (PDF 43 kb)

Additional file 3: Invitation to participate. (PDF 197 kb)

## Data Availability

The data used and/or analyzed during the current study are available from the corresponding author on reasonable request.

## Abbreviations

COS: Core outcome set
FGR: Fetal growth restriction
GRN: Growth restricted newborn
MRS: Minimum reporting set
NEC: Necrotizing enterocolitis
NICU: Neonatal intensive care unit
