# Supplementary material for: Development of a Core Outcome Set and Minimum Reporting Set for intervention studies in growth restriction in the NEwbOrN (COSNEON): study protocol for a Delphi study
Source: Trials. 2019 Aug 17;20:511. doi: 10.1186/s13063-019-3588-9 (PMC6697910; doi:10.1186/s13063-019-3588-9)
Supplement: Supplementary file 1 — SPIRIT checklist. (PDF 99 kb) [file 13063_2019_3588_MOESM1_ESM.pdf]

SPIRIT

STANDARD PROTOCOL ITEMS: RECOMMENDATIONS FOR INTERVENTIONAL TRIALS

st: Recommended items to address in a clinical trial protocol and related documents*

Item No	Description
**mation**	
1	Descriptive title identifying the study design, population, interventions, and, if applicable, trial acronym
2a	Trial identifier and registry name. If not yet registered, name of intended registry
2b	All items from the World Health Organization Trial Registration Data Set
3	Date and version identifier
4	Sources and types of financial, material, and other support
5a	Names, affiliations, and roles of protocol contributors
5b	Name and contact information for the trial sponsor
5c	Role of study sponsor and funders, if any, in study design; collection, management, analysis, and interpretation of data; writing of the report; and the decision to submit the report for publication, including whether they will have ultimate authority over any of these activities
5d	Composition, roles, and responsibilities of the coordinating centre, steering committee, endpoint adjudication committee, data management team, and other individuals or groups overseeing the trial, if applicable (see Item 21a for data monitoring committee)
6a	Description of research question and justification for undertaking the trial, including summary of relevant

studies (published and unpublished) examining benefits and harms for each intervention

6b Explanation for choice of comparators

7 Specific objectives or hypotheses

8 Description of trial design including type of trial (eg, parallel group, crossover, factorial, single group), allocation ratio, and framework (eg, superiority, equivalence, noninferiority, exploratory)

ts, interventions, and outcomes

9 Description of study settings (eg, community clinic, academic hospital) and list of countries where data will be collected. Reference to where list of study sites can be obtained

10 Inclusion and exclusion criteria for participants. If applicable, eligibility criteria for study centres and individuals who will perform the interventions (eg, surgeons, psychotherapists)

11a Interventions for each group with sufficient detail to allow replication, including how and when they will be administered

11b Criteria for discontinuing or modifying allocated interventions for a given trial participant (eg, drug dose change in response to harms, participant request, or improving/worsening disease)

11c Strategies to improve adherence to intervention protocols, and any procedures for monitoring adherence (eg, drug tablet return, laboratory tests)

11d Relevant concomitant care and interventions that are permitted or prohibited during the trial

12 Primary, secondary, and other outcomes, including the specific measurement variable (eg, systolic blood pressure), analysis metric (eg, change from baseline, final value, time to event), method of aggregation (eg median, proportion), and time point for each outcome. Explanation of the clinical relevance of chosen efficacy and harm outcomes is strongly recommended

13 Time schedule of enrolment, interventions (including any run-ins and washouts), assessments, and visits fc participants. A schematic diagram is highly recommended (see Figure)

14 Estimated number of participants needed to achieve study objectives and how it was determined, including clinical and statistical assumptions supporting any sample size calculations

15 Strategies for achieving adequate participant enrolment to reach target sample size

nt of interventions (for controlled trials)

16a	Method of generating the allocation sequence (eg, computer-generated random numbers), and list of any factors for stratification. To reduce predictability of a random sequence, details of any planned restriction (eg, blocking) should be provided in a separate document that is unavailable to those who enrol participant[illegible] or assign interventions
16b	Mechanism of implementing the allocation sequence (eg, central telephone; sequentially numbered, opaque, sealed envelopes), describing any steps to conceal the sequence until interventions are assigned
16c	Who will generate the allocation sequence, who will enrol participants, and who will assign participants to interventions
17a	Who will be blinded after assignment to interventions (eg, trial participants, care providers, outcome assessors, data analysts), and how
17b	If blinded, circumstances under which unblinding is permissible, and procedure for revealing a participant’s allocated intervention during the trial

ction, management, and analysis

18a	Plans for assessment and collection of outcome, baseline, and other trial data, including any related processes to promote data quality (eg, duplicate measurements, training of assessors) and a description of study instruments (eg, questionnaires, laboratory tests) along with their reliability and validity, if known. Reference to where data collection forms can be found, if not in the protocol
18b	Plans to promote participant retention and complete follow-up, including list of any outcome data to be collected for participants who discontinue or deviate from intervention protocols
19	Plans for data entry, coding, security, and storage, including any related processes to promote data quality (eg, double data entry; range checks for data values). Reference to where details of data management procedures can be found, if not in the protocol
20a	Statistical methods for analysing primary and secondary outcomes. Reference to where other details of the statistical analysis plan can be found, if not in the protocol
20b	Methods for any additional analyses (eg, subgroup and adjusted analyses)

20c	Definition of analysis population relating to protocol non-adherence (eg, as randomised analysis), and any statistical methods to handle missing data (eg, multiple imputation)

g

21a	Composition of data monitoring committee (DMC); summary of its role and reporting structure; statement of whether it is independent from the sponsor and competing interests; and reference to where further details about its charter can be found, if not in the protocol. Alternatively, an explanation of why a DMC is not needed
21b	Description of any interim analyses and stopping guidelines, including who will have access to these interim results and make the final decision to terminate the trial
22	Plans for collecting, assessing, reporting, and managing solicited and spontaneously reported adverse events and other unintended effects of trial interventions or trial conduct
23	Frequency and procedures for auditing trial conduct, if any, and whether the process will be independent from investigators and the sponsor

ation

24	Plans for seeking research ethics committee/institutional review board (REC/IRB) approval
25	Plans for communicating important protocol modifications (eg, changes to eligibility criteria, outcomes, analyses) to relevant parties (eg, investigators, REC/IRBs, trial participants, trial registries, journals, regulators)
26a	Who will obtain informed consent or assent from potential trial participants or authorised surrogates, and how (see Item 32)
26b	Additional consent provisions for collection and use of participant data and biological specimens in ancillary studies, if applicable
27	How personal information about potential and enrolled participants will be collected, shared, and maintained in order to protect confidentiality before, during, and after the trial
28	Financial and other competing interests for principal investigators for the overall trial and each study site
29	Statement of who will have access to the final trial dataset, and disclosure of contractual agreements that

	limit such access for investigators
30	Provisions, if any, for ancillary and post-trial care, and for compensation to those who suffer harm from trial participation
31a	Plans for investigators and sponsor to communicate trial results to participants, healthcare professionals, the public, and other relevant groups (eg, via publication, reporting in results databases, or other data sharing arrangements), including any publication restrictions
31b	Authorship eligibility guidelines and any intended use of professional writers
31c	Plans, if any, for granting public access to the full protocol, participant-level dataset, and statistical code
32	Model consent form and other related documentation given to participants and authorised surrogates
33	Plans for collection, laboratory evaluation, and storage of biological specimens for genetic or molecular analysis in the current trial and for future use in ancillary studies, if applicable

ended that this checklist be read in conjunction with the SPIRIT 2013 Explanation & Elaboration for important clarifi
rotocol should be tracked and dated. The SPIRIT checklist is copyrighted by the SPIRIT Group under the Creative C
iercial-NoDerivs 3.0 Unported" license.
