# Supplementary material for: Development of a Core Outcome Set and Minimum Reporting Set for intervention studies in growth restriction in the NEwbOrN (COSNEON): study protocol for a Delphi study
Source: Trials. 2019 Aug 17;20:511. doi: 10.1186/s13063-019-3588-9 (PMC6697910; doi:10.1186/s13063-019-3588-9)
Supplement: Supplementary file 2 — COS-STAP checklist. (PDF 43 kb) [file 13063_2019_3588_MOESM2_ESM.pdf]

COS-STAP COSNEON

TITLE/ABSTRACT			Page
Title	1a	Identify in the title that the paper describes the protocol for the planned development of a COS	1
Abstract	1b	Provide a structured abstract	2, 3
INTRODUCTION			
Background and objectives	2a	Describe the background and explain the rationale for developing the COS, and identify the reasons why a COS is needed and the potential barriers to its implementation	3, 4
	2b	Describe the specific objectives with reference to developing a COS	4
Scope	3a	Describe the health condition(s) and population(s) that will be covered by the COS	7
	3b	Describe the intervention(s) that will be covered by the COS	7
	3c	Describe the context of use for which the COS is to be applied	7

METHODS			
Stakeholders	4	Describe the stakeholder groups to be involved in the COS development process, the nature of and rationale for their involvement and also how the individuals will be identified; this should cover involvement both as members of the research team and as participants in the study	8, 9
Information sources	5a	Describe the information sources that will be used to identify the list of outcomes. Outline the methods or reference other protocols/papers	7
	5b	Describe how outcomes may be dropped/combined, with reasons	9, 10, 11, 12
Consensus process	6	Describe the plans for how the consensus process will be undertaken	9, 10, 11, 12
Consensus definition	7a	Describe the consensus definition	10, 11, 12
	7b	Describe the procedure for determining how outcomes will be added/combined/dropped from consideration during the consensus process	9, 10, 11, 12
ANALYSIS			

Outcome scoring/feedback	8	Describe how outcomes will be scored and summarised, describe how participants will receive feedback during the consensus process	9, 10
Missing data	9	Describe how missing data will be handled during the consensus process	10
ETHICS and DISSEMINATION			
Ethics approval/informed consent	10	Describe any plans for obtaining research ethics committee/institutional review board approval in relation to the consensus process and describe how informed consent will be obtained (if relevant)	8, 9, 13, additional file 3
Dissemination	11	Describe any plans to communicate the results to study participants and COS users, inclusive of methods and timing of dissemination	12
ADMINISTRATIVE INFORMATION			
Funders	12	Describe sources of funding, role of funders	13
Conflicts of interest	13	Describe any potential conflicts of interest within the study team and how they will be managed	13
