# Supplementary material for: Development of a Core Outcome Set and Minimum Reporting Set for intervention studies in growth restriction in the NEwbOrN (COSNEON): study protocol for a Delphi study
Source: Trials. 2019 Aug 17;20:511. doi: 10.1186/s13063-019-3588-9 (PMC6697910; doi:10.1186/s13063-019-3588-9)
Supplement: Supplementary file 3 — Invitation to participate. (PDF 197 kb) [file 13063_2019_3588_MOESM3_ESM.pdf]

Invitation to participate

Thank you for showing an interest in helping us to improve research and care in children born too small.

Over the next six months, we will be developing a **'minimum reporting set'**, concerning reporting items of a study population, and a **'core outcome set'** concerning outcomes of a study. The main aim is for researchers to use them in order to improve the quality of research around care for babies born too small following fetal growth restriction. Please click this video for a short explanation.

What is an outcome? What are reporting items?

In deciding on interventions and treatments for children born too small, parents and health professionals need evidence about what interventions and treatments are most effective. Health professionals get this evidence by undertaking research to measure the effects of interventions and treatments. These effects are called 'outcomes' and outcomes cover a wide range of different effects; for example, the number of days a child needs to be in hospital, how quickly children grow and how he/she meets developmental milestones after leaving hospital.

Reporting items concern other important elements that define a study and its population. For example gestational age at birth or the type of intervention that is used to treat a baby.

What is a core outcome set? What is a minimum reporting set?

A Core Outcome Set (COS) is a minimum set of outcomes that needs to be measured when investigating a certain topic (in this case, intrevention and treatment strategies for growth restricted babies). A Core Outcome Set makes it much more straightforward to compare different studies in the same area. Different studies can only be compared if the same effects/outcomes are measured in each study. For example: if one study measures growth over time (grams per day) and the other study measures days in the neonatal intensive care unit or infection rates, we cannot compare the results. This hampers the choice of interventions and treatments. To address this issue, researchers have

started to develop core outcome sets. Core outcome sets are a list of important outcomes that parents of patients and health professionals have decided on together. The idea is that when a core outcome set is developed, all the research teams working on the same condition will then use the core outcome set in their research. This will make it possible to compare treatments across many studies, which will help guide patients, parents and health professionals in deciding on the best interventions and treatments. Similar to a core outcome set, a minimum reporting set (MRS) is a minimum set of items that should be reported to properly define the study population. The same principles apply.

For more information about a Core Outcome Set in general, we would like to refer you to this video (Comet Initiative, 2018).

How will we develop a core outcome set and a minimum reporting set?

To decide which outcomes and baseline characteristics are most important, researchers need to get the opinion of all relevant stakeholders and try to reach agreement, or 'consensus', on the most important outcomes and baseline characteristics.

We are inviting groups of 'experts' in different groups of stakeholders, including parents of patients, health professionals and researchers who have experience with fetal growth-restriction to take part in this process. For this study, we will be inviting people from all over the world. We will ask them their opinions on what outcomes and reporting items are most important to them - this is anonymous to make sure everyone has an equal vote.

We need all stakeholders to complete three online surveys over the next few months. Each online survey will take approximately 10-15 minutes. It is really important that you complete all three surveys.

IMPORTANT!! we look for a **MINIMUM set of outcomes** and items to be reported in future studies, and we do not look for ALL important issues in intervention strategies for babies with fetal growth restriction. Individual studies may still choose to report additional outcomes and items to report to those in the agreed Core Outcome Set and Minimum Reporting Set. So please choose carefully and be exclusive rather than inclusive with the items.

Use of your personal data

Personal data (your name and email address) will only be used for study purposes and data will be analysed on group level: parents/lay experts, clinicians/researchers. No answer can be related to you as a person by anyone other than the research group. You can always withdraw your participation; however, responses from previous rounds will remain stored (due to the logistics of Delphi procedures). Unless you withdraw, your personal details will be saved as long as required by national research laws.

By subscribing to this procedure and entering your name and email address you agree to these details and to participate in the study.

Who can I contact if I have any questions?

If you have any questions, please contact s.j.gordijn@umcg.nl

How do I take part and help with the project?

If you are interested in helping us to develop the core outcome set and minimum reporting set, we would be very grateful if you could spend two minutes completing a registration questionnaire. Once you have completed the registration questionnaire, we will email you within a few weeks about the first phase of the online survey.

Kind regards,

Frank Bloomfield (neonatologist, developer of COS FGR), Sanne Gordijn, Asma Khalil, Wessel Ganzevoort (obstetricians developers of COS on FGR), Tabitha Piet, Martine Knol, Helena Wang, (bachelor students), Stefanie Damhuis (master student), Mandy Daly (patient representative),

on behalf of the COSNEON team
